# 

**Published:** 1885-03

**Authors:** 


### CONTENTS.

### COMMUNICATIONS.

Treatment and Filling of the Nerve Canals in Teeth .......... 109
Description of Furnace .......... 119

### SELECTIONS.

Sponge Grafting .......... 122
Diseases of the Teeth in Relation to So-Called Reflex Maladies .......... 126
Again a Sale of American Diplomas in Europe .......... 132
Collective Investigation of Dental Diseases .......... 136
Cocaine .......... 138
Aseptol, the New Antiseptic .......... 141
Plastic Operations on the Palate .......... 142
Hamamelis in Epistaxis of Typhoid Fever .......... 143
Hydrochlorate of Cocaine .......... 144
Heart Beats .......... 145
Glycerine as an Excipient .......... 146
Cocaine as an Anæsthetic and Analgetic for Pharynx and Larynx .......... 147
Periostitis Following Typhoid Fever .......... 148
CORRESPONDENCE .......... 149
SALMAGUNDI .......... 150

### EDITORIAL.

Diseased Maxillary Sinus .......... 155
Legislation .......... 156
National Association of Dental Examiners .......... 157
Southern Dental Association .......... 158
Ohio State Dental Society—Re-organized .......... 159
Meeting .......... 160
Necrology .......... 160

